# MicroRNA Expression in Breast Cancer Patients, an Integrative Review

**DOI:** 10.1002/jso.70132

**Published:** 2025-11-16

**Authors:** Larissa Fernanda Campos Moreira da Silva, Fabiana Pirani Carneiro, Andrea Barretto Motoyama

**Affiliations:** ^1^ School of Medicine University of Brasília (UnB), Military Hospital of the Brasília Area Brasília Federal District Brazil; ^2^ University of São Paulo (USP) São Paulo São Paulo Brazil; ^3^ School of Medicine University of Brasília (UnB) Brasília Federal District Brazil; ^4^ University of Basel Basel Switzerland; ^5^ Faculty of Medicine University of Brasília (UnB) Brasília Federal District Brazil; ^6^ Faculty of Health Sciences University of Brasília (UnB) Brasília Federal District Brazil

**Keywords:** breast cancer, gene expression, integrative review, microRNA, molecular biomarkers

## Abstract

This integrative review examines the differential expression of miRNAs in breast cancer patients across various disease stages. Through analysis of 42 articles published between 2005 and 2023, the review identifies multiple miRNAs with significant diagnostic, prognostic, and predictive potential in breast cancer management. These small noncoding RNAs, accessible through liquid biopsy, demonstrate notable potential for early detection, molecular subtype classification, treatment response prediction, and relapse monitoring. The findings highlight miRNAs as promising biomarkers for precision medicine approaches, potentially enhancing clinical decision‐making and improving patient outcomes. Notably, miRNAs such as miR‐21, miR‐155, and members of the miR‐200 family show consistent associations with clinical parameters across multiple studies. The accessibility of miRNAs in blood and other body fluids, combined with their stability and specificity, positions them as valuable tools that could complement conventional diagnostic methods and support more personalized treatment strategies in breast cancer care.

## 1 Introduction

Breast cancer is the most common malignancy in women, accounting for 12.5% of all cancers and the leading cause of cancer‐related death in the Western world [1, 2]. Despite advances in screening and treatment, over 50% of patients experience relapse [3]. The disease is multifactorial, influenced by both genetic and environmental factors. Identifying women at high risk of recurrence and those who may benefit from adjuvant therapy is crucial. Prognostic factors predict the risk of recurrence or death, while predictive factors identify patients likely to respond to specific treatments, enabling personalized therapeutic strategies [4, 5, 6].

Breast tumors are classified into subtypes based on gene expression, which guides therapy selection [6, 7]. Recent advancements in gene expression profiling have identified genetic signatures as noninvasive biomarkers for prognosis and treatment prediction [8]. Molecular subtyping, as shown by Sørlie et al. [9], provides more accurate prognostic stratification. Their study found that luminal A and B (mostly ER + ) tumors had higher survival rates, while triple‐negative (ER−, PR−, HER2−) tumors had worse outcomes, with survival times under 48 months. These findings support integrating molecular signatures with histopathological classification to enhance prognosis and treatment stratification.

The eighth edition of the AJCC TNM staging system incorporates Tumor (T) size, Regional Lymph (N), metastasis (M), histological grade, hormone receptor status, Her2 protein expression, and genomic profiles. However, it still lacks precision in predicting long‐term prognosis, as many patients relapse after several years [6, 7, 8, 9, 10]. This highlights the need for biomarkers to monitor treatment, disease progression, and relapse. This underscores the need for reliable biomarkers to monitor treatment response, disease progression, and recurrence.

MicroRNAs (miRNAs) are small noncoding RNAs (18–22 nucleotides) that regulate gene expression and can be found both intracellularly and extracellularly (in plasma, urine, and saliva). Their accessibility and stability make miRNAs promising biomarkers for breast and other cancers. Liquid biopsy, which detects biomolecules like miRNAs in body fluids, offers a noninvasive and accurate approach to monitor diagnosis, prognosis, treatment, and disease progression [1, 2, 3]. This integrative review aims to summarize current evidence on miRNAs as biomarkers in breast cancer diagnosis, prognosis, and treatment monitoring.

## 2 Materials and Methods

This integrative literature review addresses the question: “Can different levels of microRNA in breast cancer patients at various disease stages be used for diagnosis, prognosis, stage definition, treatment response monitoring, and relapse?”

The **‘PICOT’** strategy was applied as follows:
**P:** Female patients diagnosed with breast cancer.
**I:** Not applicable, since all clinically accepted interventions were taken into consideration.
**C:** Comparisons between patient groups with different clinical outcomes (e.g., recurrence vs. no recurrence, response vs. nonresponse to treatment, early vs. advanced stage).
**O:** Association of miRNA levels with clinical events and their predictive/prognostic value.
**T:** Original studies (exploratory, descriptive, retrospective, or prospective) with a (semi‐) quantitative approach, published between 2005 and 2023.

Since this is an integrative literature review rather than a clinical trial, a specific intervention (I) is not applicable. The review instead considers all standard‐of‐care treatments to assess miRNA expression across different clinical contexts

The literature search was conducted in LILACS, MEDLINE (via PubMed), SciELO, Web of Science, Scopus, and EBSCOHost, using Boolean operators “AND” and “OR”. Keywords and DeCS terms included: Breast Neoplasms, Breast Neoplasia, Predictive Value of Tests, and Prognostic Factors, along with MeSH terms: Breast Neoplasia, microRNAs, Predictive Value of Tests, and Prognostic Factors.

Inclusion criteria were full texts in Portuguese, English, or Spanish, focused on women over 19 years, and published between 2005 and 2023. Exclusion criteria were gray literature, books, conference proceedings, letters to editors, case reports, reviews, and poster abstracts. Studies were first selected by title and abstract, then read in full, resulting in 42 articles.

A bootstrap approach was used, where literature cited by the selected articles was also checked and included if it met the criteria. Studies using exosomal miRNA were excluded, as the focus was on free circulating miRNAs.

## 3 Results

### 3.1 Studies Characteristics

After complete evaluation, 41 studies were included herein (shown in Table 1). Most of the studies were published in 2019 and 2022 (65%, 85%), and all were in English. The studies were conducted in countries such as China, Germany, Greece, Italy, Iran, and Pakistan, with most involving fewer than 100 patients.

**Table 1:** Summary of studies included in the integrative review on microrna expression in breast cancer patients (*n *= 42).

Title	Authors	Year	Main finding
Identification of a two‐microrna signature in plasma as a novel biomarker for very early diagnosis of breast cancer	Adam‐Artigues [11]	2021	miR‐30b‐5p and miR‐99a‐5p as early diagnostic biomarkers
Decoding the usefulness of noncoding RNAs as breast cancer markers.	Amorim [12]	2016	ncRNAs are promising for diagnosis and treatment of breast cancer
Predictive and Prognostic Value of Selected MicroRNAs in Luminal Breast Cancer.	Amorim [13]	2019	miR‐30c‐5p, miR‐30b‐5p, miR‐182‐5p, miR‐200b‐3p predict response in luminal BC
Relative and Absolute Expression Analysis of MicroRNAs Associated with Luminal A Breast Cancer—A Comparison	Arabkari [14]	2020	miR‐145, miR‐195, miR‐486 best diagnostic for luminal A BC; normalization by miR‐16 may vary
Combination of circulating miR‐145‐5p/miR‐191‐5p as biomarker for breast cancer detection	Ashirbekov [15]	2020	miR‐145‐5p/miR‐191‐5p as plasma diagnostic biomarkers in Kazakh women
Evaluation of plasma miR‐21 and miR‐152 as diagnostic biomarkers for common types of human cancers	Chen [16]	2016	miR‐21 and miR‐152 have diagnostic potential in lung, colorectal, and breast cancer
A three‐microRNA signature predicts clinical outcomes in breast cancer patients.	Cheng [17]	2018	Three‐miRNA signature may be prognostic for invasive breast cancer
Investigation of BRCAness associated miRNA‐gene axes in breast cancer: cell‐free miR‐182‐5p as a potential expression signature of BRCAness	Darbeheshti [18]	2022	Plasma miR‐182‐5p identifies BRCAness; potential for PARP inhibitor eligibility
Plasma miRNA levels for predicting therapeutic response to neoadjuvant treatment in HER2‐positive breast cancer: Results from the NeoALTTO trial	Di Cosimo [19]	2019	ct‐miRNAs can predict trastuzumab therapy response in HER2+ patients
Early modulation of circulating microRNAs levels in HER2‐positive breast cancer patients treated with trastuzumab‐based neoadjuvant therapy	Di Cosimo [20]	2020	Increased miR‐148a‐3p/miR‐374a‐5p linked to complete response to trastuzumab
End‐of‐neoadjuvant treatment circulating microRNAs and HER2‐positive breast cancer patient prognosis: An exploratory analysis from NeoALTTO	Di Cosimo [21]	2023	Three‐miRNA signature prognostic in HER2+ post‐neoadjuvant trastuzumab
Early detection breast cancer: Role of circulating plasma miRNA‐21 expression as a potential screening biomarker	Diansyah [22]	2021	Circulating miR‐21 is a potential early detection biomarker
Plasma microRNA pair panels as novel biomarkers for detection of early stage breast cancer	Fang [23]	2019	Plasma miRNA pair panels are novel early detection biomarkers
Circulating microRNA‐based screening tool for breast cancer	Frères [24]	2016	Circulating microRNA panel as noninvasive screening tool for early detection
Total circulating microRNA level as an independent prognostic marker for risk stratification in breast cancer	Gahlawat [25]	2022	Total circulating miRNA level is a prognostic marker
Analysis of plasma miRNA‐497 levels in the blood of patients with breast cancer	Garashchenko [26]	2023	Plasma miR‐497 level relates to molecular subtype and stage
Plasma mir‐1273g‐3p acts as a potential biomarker for early breast ductal cancer diagnosis	Guo [27]	2020	Plasma miR‐1273g‐3p for early ductal BC diagnosis; correlates with TNM stage
Plasma exosome microRNAs are indicative of breast cancer	Hannafon [28]	2016	Exosomal miR‐21 and miR‐1246 elevated in breast cancer plasma
Assessment of circulating miRNA levels in breast cancer patients depending on clinical characteristics and chemotherapy	Harashchenko [29]	2023	miR‐25, miR‐27, miR‐335, miR‐497 as potential markers for treatment efficacy
Pathway analysis of selected circulating miRNAs in plasma of breast cancer patients: A preliminary study	Holubekova [30]	2020	miR‐99a, miR‐130a, miR‐484, miR‐1260a help discriminate BC patients from controls
Appraising microRNA‐155 as a noninvasive diagnostic biomarker for cancer detection: A meta‐analysis	Hou [31]	2016	miR‐155 is a strong noninvasive diagnostic biomarker
MicroRNA‐21 promotes breast cancer proliferation and metastasis by targeting LZTFL1	Hui Wang [32]	2019	miR‐21 promotes proliferation/metastasis via LZTFL1 targeting
Circulating miRNAs in untreated breast cancer: An exploratory multimodality morpho‐functional study	Incoronato [33]	2019	miR‐125b‐5p, miR‐143‐3p upregulated in BC, distinguish from controls
MicroRNA gene expression deregulation in human breast cancer.	Iorio [34]	2005	miRNAs deregulated in breast cancer, play key pathogenetic roles
Multiple microRNAs as biomarkers for early breast cancer diagnosis	Jang [3]	2021	miR‐1246, miR‐206, miR‐24, miR‐373: 98% sensitivity, 96% specificity for BC detection
MicroRNA in diagnosis and therapy monitoring of early‐stage triple‐negative breast cancer	Kahraman [35]	2018	miRNAs in blood aid early TNBC diagnosis; may predict complete response
A novel six‐microRNA‐based model to improve prognosis prediction of breast cancer.	Lai [36]	2019	Six‐miRNA model is reliable for survival prediction
A five‐miRNA panel in plasma was identified for breast cancer diagnosis	Li [37]	2019	Five plasma miRNAs identified as BC diagnostic biomarkers
MiR‐210 and miR‐152 as biomarkers by liquid biopsy in invasive ductal carcinoma	Lopes [38]	2021	miR‐210, miR‐152 are noninvasive biomarkers, relate to staging and angiogenesis
Direct Comparison of Metastasis‐Related miRNAs Expression Levels in Circulating Tumor Cells, Corresponding Plasma, and Primary Tumors of Breast Cancer Patients	Markou [39]	2022	miR‐21, miR‐146a, miR‐200c, miR‐210 valuable for metastatic BC detection
Tumor microRNA expression profiling identifies circulating microRNAs for early breast cancer detection.	Matamala [40]	2015	miRNA signatures distinguish BC from healthy tissue with high sensitivity and specificity
Circulating microRNAs miR‐331 and miR‐195 differentiate local luminal A from metastatic breast cancer	McAnena [41]	2019	miR‐331 differentiates metastatic from local luminal A BC
Circulating microRNAs as specific biomarkers for breast cancer detection.	Ng [42]	2013	Circulating miRNAs (miR‐16, miR‐21, etc.) as BC screening markers
Circulating miRNAs as a marker of metastatic disease and prognostic factor in metastatic breast cancer	Papadaki [43]	2019	High miR‐200b predicts shorter survival in metastatic BC
MicroRNAs regulating tumor and immune cell interactions in the prediction of relapse in early‐stage breast cancer	Papadaki [44]	2021	Immunomodulatory miRNAs in plasma predict relapse in early BC
What if the future of HER2‐positive breast cancer patients was written in miRNAs? An exploratory analysis from NeoALTTO study.	Pizzamigli [45]	2022	Tumor miRNA expression profile has prognostic/predictive value in trastuzumab‐treated HER2+
Circulating plasma miRNA‐21 as a superior biomarker compared to CA 15‐3: Assessment in healthy age‐matched subjects and different stage of breast cancer patients	Savitri [46]	2020	miR‐21 superior to CA 15‐3 for BC diagnosis and stage detection
Identification of circulating miRNAs as noninvasive biomarkers of triple‐negative breast cancer in the population of Pakistan	Shaheen [47]	2019	miRNA panel (miR‐376c, miR‐155, miR‐17a, miR‐10b) for TNBC diagnosis and staging
Role of some circulating miRNAs on breast cancer diagnosis.	Swellam [48]	2019	miR‐17‐5p, miR‐155, miR‐222 superior to classical markers for early BC diagnosis
MicroRNAs Regulating Tumor Immune Response in the Prediction of the Outcome in Patients With Breast Cancer	Thomopoulou [49]	2021	miR‐10b, miR‐19a, miR‐20a, miR‐126, miR‐155 predict outcomes in BC
A circulating miR‐19b‐based model in diagnosis of human breast cancer	Zhao [50]	2022	Circulating miR‐19b‐based model: 92% sensitivity, 90% specificity

Abbreviations: AUC, area under the curve; BC, breast cancer; miRNA, microRNA; ncRNA, noncoding RNA; pCR, pathological complete response; RT‐qPCR, real‐time quantitative polymerase chain reaction.

Nearly all studies assessed a limited number of miRNAs. Some started with a smaller discovery cohort of patients to identify miRNA signatures and then used a larger cohort for confirmation [4, 39]. One study [14] did not focus on specific miRNAs but assessed total miRNA levels in a large population (250 breast cancer patients and 356 healthy individuals) over 9 years, proposing miRNAs for prognosis.

### 3.2 MiRNAs for Diagnosis

MiRNAs have been widely tested for complementary diagnosis, as they can differentiate breast cancer patients from healthy individuals [7, 15, 25, 26, 28, 31, 41, 42, 43]. In some cases, using miRNA for diagnosis resulted in high specificity and sensitivity [24, 28, 36, 39]. Some studies used miRNAs to discriminate specific types of breast cancer, such as luminal A [7], HER2+ [42, 43], TNBC [26], and metastatic [15]. Lymph node infiltration was also evaluated [42]. Savitri et al. (2020) [45] compared the expression of plasma miR‐21 and CA 15‐3 (a clinically used biomarker) and found that miR‐21 could be a superior biomarker for breast cancer detection.

In fact, of all miRNAs, miR‐21 is the most frequently associated with breast cancer. One study [16] investigated the correlation between miR‐21 and miR‐206 levels with estrogen receptors (ER), progesterone receptor (PR), and human epidermal growth factor receptor 2 (HER2). MiR‐21 levels were positively correlated with ER and PR expression, while miR‐206 levels were negatively correlated with HER2 expression. MiR‐21 also promoted breast cancer proliferation and metastasis by targeting the LZTFL1 gene.

### 3.3 MiRNAs for Prognosis

MiRNAs have also been investigated as prognostic markers, either generally or in response to specific treatments. Kahraman et al. (2018) [3] studied basal‐like TNBC patients and found that measuring miRNAs as early as 2 weeks into neoadjuvant chemotherapy could predict pathological complete response (pCR). Amorim et al. (2019) [13] identified two miRNA signatures: the first one (miR153‐3p and miR219‐5p) significantly related to event‐free survival and a second one (miR‐215‐5p and miR30c‐2‐3p) significantly associated with pathological response in luminal BC. Papadaki et al. (2019) [42] measured miRNA levels in early and metastatic BC and showed that miR‐200b could independently predict shorter overall survival (OS) in metastatic BC. Amorim et al. (2016) [12] showed that miR30c‐5p, miR30b‐5p, miR182‐5p, and miR200b‐3p expression levels independently predicted endocrine resistance‐free survival. Di Cosimo et al. (2020, 2023) [19, 20, 21] determined that miR‐148a‐3 and miR‐374a‐5p were significantly associated with pCR in HER2+ patients undergoing trastuzumab‐based neoadjuvant therapy.

Additionally, Lai et al. [23] constructed a six‐miRNA prognostic model composed of hsa‐miR‐549a, hsa‐miR‐6715a, hsa‐miR‐4501, hsa‐miR‐7974, hsa‐miR‐4675 (associated with increased risk) and hsa‐miR‐147b (protective). This signature demonstrated a good ability to predict 5‐year overall survival, with an AUC of 0.701 in the training cohort and 0.789 in the validation cohort. When integrated into a nomogram with clinical variables such as age, TNM stage and ER status, the predictive performance further improved, reaching an AUC of 0.758 and 0.777, respectively. Two other groups were able to show the prognostic value of miRNAs, albeit in more restricted populations. Amorim et al. [3, 4] demonstrated that specific miRNAs (miR‐153‐3p, miR‐219‐5p, miR‐215‐5p, miR‐30c‐2‐3p) were linked to event‐free survival and response to endocrine therapy, especially in luminal BC, while Papadaki et al. [32, 33] worked with metastatic BC patients and found that high miR‐200b correlated with shorter survival.

MiRNAs for monitoring treatment and minimal residual dPathological response is an objective feature that can be used to monitor therapy response and can also be correlated to miRNAs levels. Many of the studies adressing pR(pCR) concurrently looked at pronognosis. Working with different populations, Kahraman et al. (2018) [3], Amorim et al. 2019 [13], and Di Cosimo independently showed that miRNAs could predict pathological response. Their studies were presented in the former subsection (prognosis).

## 4 Discussion

An extensive literature review was conducted on the use of microRNAs (miRNAs) as biomarkers in breast cancer (BC), focusing on their potential for diagnosis, prognosis, and prediction of therapeutic response. The majority of studies [7, 13, 15, 16, 22, 23, 25, 28, 31], employed a case‐control cohort design, which is considered a reliable standard by the Agency for Healthcare Research and Quality (AHRQ) [33, 34, 39, 42, 44, 45, 47].

Overall, miRNAs were more frequently used for diagnosis than for prognosis or therapeutic response and showed themselves to be more sensitive and more reliable biomarkers for that end. Studies demonstrated that miRNAs had significant diagnostic power for BC [1, 2, 3, 6, 7, 12, 15, 17, 18, 20, 21, 22, 23, 24, 26, 27, 28, 30, 31, 36, 37, 39, 41, 42, 45, 47, 50, 51, 52, 53]. Whether using one single miRNA, a combination of different miRNAs, or a signature of miRNAs and additional markers such as proteins, the panels studied showed high sensitivity, specificity and accuracy levels ‐ usually beyond 90% in each study [24, 36] or at least over 80% [39]. Such diagnostic power may be forwarded due to the fact that traditional methods for BC diagnosis are currently reliable and relatively straightforward, with well‐known markers and features that can be used for direct comparison. In this sense, it is interesting to observe that miRNAs (specifically miR‐21) were also validated in comparison to other markers, such as CA‐15‐3 [45, 53]. This type of study highlights the power of miRNAs, especially when classical serological markers or tissue biopsies are inconclusive or impractical.

The prognostic value of biomarkers usually requires longer follow‐up times, as well as larger cohorts. One attempt to circumvent these barriers was put forward by Lai et al. [23], who proposed a theoretical model based on a six‐miRNA signature (hsa‐miR‐549a, hsa‐miR‐6715a, hsa‐miR‐4501, hsa‐miR‐7974, hsa‐miR‐4675, and hsa‐miR‐147b). This model showed reliable prediction of 5‐year overall survival, with an AUC of 0.701 in the training cohort and 0.789 in the validation cohort; when combined with clinical variables in a nomogram, the AUC reached 0.758 and 0.777, respectively. Despite these promising results, the model still requires experimental validation. More restricted analyses—perhaps easier to conduct—have also demonstrated the prognostic value of miRNAs. Amorim et al. [3, 4] reported that specific miRNAs (miR‐153‐3p, miR‐219‐5p, miR‐215‐5p, miR‐30c‐2‐3p) were associated with event‐free survival and response to endocrine therapy, particularly in luminal BC, while Papadaki et al. [32, 33] showed that high miR‐200b expression correlated with shorter survival in metastatic cases. Circulating miRNAs hold promise for predicting and evaluating response to therapy in BC [3, 19, 20, 21, 34, 38, 43, 44].

When pathological complete response was used as a parameter, miRNAs showed early predictive value [8] for TNBC patients undergoing neoadjuvant therapy. Similarly, Di Cosimo et al. [10, 11, 54] and Pizzamiglio et al. [3] showed that specific circulating miRNAs (e.g., miR‐148a‐3p, miR‐374a‐5p, miR‐185‐5p, miR‐146a‐5p, miR‐22‐3p) predicted complete response or prognosis in HER2+ patients undergoing trastuzumab‐based regimens. Papadaki et al. [33, 34] reported that deregulation of circulating immunomodulatory miRNAs predicted relapse and outcomes in early BC. It is also expected that studies correlating miRNAs to response to therapy should emerge in the next years. However, it should be considered that this type of study is the one that must include more variables in the analyses. Not only BC type and tumor staging should be taken into consideration, but also the kind, type, dosage and duration of therapy(‐ies) could account for differences in response. Because of these limitations, it is expected that studies addressing these responses to therapy will be carried on by large institutions or in a multicenter‐basis.

One important aspect for using miRNA in liquid biopsy, whether for diagnosis or prognosis and response, is the standardization of extraction and detection methods. More commonly, arrays, NGS, RT‐qPCR, and ddPCR are used, but the chosen method may greatly influence the results. Whereas arrays and NGS can be used for discovery and large datasets, RT‐qPCR and ddPCR are more useful for data confirmation and for reduced data sets (especially when the number of distinct miRNAs to be analyzed is considered). Additionally, when working with plasma miRNA and RT‐qPCR, it is important to consider the choice of normalizing gene. Since miRNAs do not come from a specific tissue, normalization may represent a source of bias. Some studies have used miR‐16 for normalization, because it is a very abundant miRNA in plasma, while non‐BC studies have used RU6B, an enzyme that is considered to remain unchanged in plasma. When algorithms that can minimize the problem of normalization are applied, it is usually required to use several normalizing miRNAs, which can be economically challenging, especially in the clinical setting.

One key point for biomarkers, and especially for miRNA detection in plasma, is sampling quality. It is usually recommended that bobod and plasma samples are kept cold and lysis‐ and cloth‐free until sample processing. As for miRNA isolation, several methods have been tested, including those more “hands‐on” such as phenol/chloroform‐based ones and those based on magnetic‐ or column‐based separation. Although there seem not to be great differences in RNA quality among methods, total RNA recovery may vary greatly, which may represent a problem when trying to measure a “rare” miRNA. So far, as normalization has not been achieved, it is advisable that each laboratory should take into consideration all the technical issues that can impact results when conducting research on miRNA levels, and even more so, when evaluating miRNAs for clinical purposes.

It is also important to emphasize that, because of the large number of studies using plasma‐derived miRNA in the latter years, this review has limited its scope to free circulating miRNA. Other important avenue of investigation is exosomal RNA. Since nucleic acids and proteins may be carried in these small vesicles, a combination of miRNA and proteins may have important biological roles that should be investigated. Due to size limitaions, exosomal studies were not included herein, which may represent a limition in this study.

### 4.1 Other Considerations

While this review covered three languages and studies from diverse geographic backgrounds (China, Germany, Iran, etc.) [1, 2, 3, 4, 5, 6, 7, 8, 9, 10, 11, 12, 13, 14, 15, 16, 17, 18, 19, 20, 21, 22, 23, 24, 25, 26, 27, 28, 29, 30, 31, 32, 33, 34, 35, 36, 37, 38, 39, 54], the exclusion of exosomal miRNA studies might limit the full scope of current research. Exosomal miRNAs offer additional, sometimes complementary, insights but require complex isolation and characterization—excluded here to minimize confounders and maintain methodological homogeneity.

In summary, this review of 41 primary studies robustly demonstrates that circulating miRNAs are viable, noninvasive biomarkers for diagnosis, subtyping, prognosis, and therapeutic monitoring in breast cancer [1, 2, 3, 4, 5, 6, 7, 8, 9, 10, 11, 12, 13, 14, 15, 16, 17, 18, 19, 20, 21, 22, 23, 24, 25, 26, 27, 28, 29, 30, 31, 32, 33, 34, 35, 36, 37, 38, 39, 54]. Panels combining multiple miRNAs consistently outperform single markers for sensitivity and specificity. However, the transition to routine clinical practice will demand rigorous standardization, analytical harmonization, and validation in larger, well‐powered prospective cohorts.

## 5 Conclusions

Plasma microRNAs (miRNAs) are emerging as promising biomarkers for breast cancer diagnosis, prognosis, and therapeutic monitoring. Their differential expression is linked to tumor progression, metastasis, and therapeutic resistance, making them key for precision oncology.

Noninvasive detection via tissue and plasma samples enhances clinical potential, offering new approaches for early detection and treatment monitoring. Multi‐miRNA panels can improve diagnostic accuracy and personalized treatments.

However, challenges remain, such as standardizing extraction, quantification, and analysis methods. Future studies and technologies, including AI, could further refine miRNA's role in clinical practice, advancing breast cancer management and improving patient outcomes.

## Synopsis

This integrative review explores how miRNA expression patterns in breast cancer patients can serve as accessible biomarkers for diagnosis, prognosis, and treatment monitoring, offering potential enhancements to personalized care across all disease stages.

## Data Availability

The data that support the findings of this study are available on request from the corresponding author. The data are not publicly available due to privacy or ethical restrictions.
